# Continuous Electroencephalogram (cEEG) Findings and Neurodevelopmental Outcomes in Neonates with Congenital Heart Disease (CHD) at 12–24 Months of Age

**DOI:** 10.1007/s10803-024-06418-y

**Published:** 2024-05-31

**Authors:** Swetha Padiyar, Neil Friedman, Elia Pestana-Knight, Linda Franic, Sarah Worley, Hany Aly

**Affiliations:** 1 Department of Neonatology, Cleveland Clinic Children’s Hospital, 9500 Euclid Ave, M-31, Cleveland, OH 44195 USA; 2 Department of Neurology, Barrow Neurological Institute at Phoenix Children’s Hospital, Phoenix, AZ USA; 3 Epilepsy Center, Cleveland Clinic, Cleveland, OH USA; 4 Department of Quantitative Health Sciences, Cleveland Clinic, Cleveland, OH USA

**Keywords:** Congenital Heart Disease (CHD), Electroencephalogram (EEG), Neurodevelopmental outcomes

## Abstract

**Objective:**

This study aims to assess the role of continuous EEG (cEEG) background patterns and duration of cross-clamp time and cardiopulmonary bypass (CPB) in children with congenital heart disease (CHD) undergoing cardiac surgery and its correlation with abnormal neurodevelopmental outcomes at 12–24 months on Bayley Scales of Infant and Toddler Development (BSID-III).

**Methods:**

This retrospective cohort study included infants with CHD and cEEG monitoring, who underwent surgery by 44 weeks gestational age.

**Results:**

34 patients were included, who were operated at median age − 7 days. Longer duration of cross- camp time was associated with poor language composite scores (LCS) (p value = 0.036). A significant association existed between severity of encephalopathy in 24-hour post-operative period and poor LCS (p value = 0.026).

**Conclusion:**

Majority of neonates with CHD have below average cognitive, language and motor composite scores on BSID-III. Longer duration of cross-clamp time and severity of encephalopathy during 24-hour post-operative EEG monitoring are associated with poor LCS.

## Introduction

Congenital heart disease (CHD) encompasses both structural and non-structural anomalies present at birth.(Hoffman & Kaplan, 2002; Reller et al., 2008) Congenital heart defects or structural malformations of the heart are the most common type of birth defects in the US with an incidence of 4–50/1000 live births reported by various studies.(Razzaghi et al., 2015) Neonates with CHD have an increased risk of brain injury. (Shillingford et al., 2008; Wernovsky et al., 2005)The etiology of brain injury in neonates who undergo corrective surgery for CHD is multifactorial.(Meyer et al., 2017) Hypothesized etiologies include disturbances in brain metabolic function, brain injury, and abnormal brain development in addition to genetic predisposition.(Ransom & Srivastava, 2007) Neurodevelopmental disability after congenital cardiac surgery is common and is the most consequential sequelae of congenital heart disease (CHD).(Shillingford et al., 2008; Wernovsky et al., 2005) Recent literature shows higher risk of neurodevelopmental impairment such as difficulties with language, attention, academic achievement, fine and gross motor skills and psychological factors in this population. (Bellinger et al., 1995; Hövels-Gürich et al., 2006; Kirshbam et al., 2005; Mahle et al., 2000) Prospective studies in infants with CHD who had undergone cardiopulmonary bypass (CPB) surgery reported cognitive outcomes within the low normal range with motor outcomes > 1 standard deviation below population means.(Bellinger et al., 1995; Goldberg et al., 2007) Mortality rate and survival outcomes in neonates with CHD undergoing surgical repair have improved over time, with advances in medical therapies, surgical techniques and critical care management. It is estimated that more than 90% of these children survive into adulthood.(Green, 2004) (Oster et al., 2013) Given the increased survival rate, focus has now shifted from preventing mortality to preventing adverse neurodevelopmental outcomes. Methods to identify brain injury and predict neurodevelopmental outcomes using non-invasive neuro-diagnostic tests such as continuous electroencephalography (cEEG) could improve prognostication and long-term monitoring in these children. The American Clinical Neurophysiology Society (ACNS) guidelines recommends long term EEG monitoring in neonates and infants with CHD that require early surgery.(Shellhaas et al., 2011) A previous study showed that only 55% of infants had normal pre-operative EEG. (C. et al., 2001) Clinical seizures during post-operative period have been described in 5–20% of neonates whereas EEG only seizures are more common occurring in 5–26% neonates.(Chock et al., 2006; Clancy et al., 2003, 2005; Gaynor et al., 2005; Gunn et al., 2012b; Helmers et al., 1997; Newburger et al., 1993) A strong predictive value of adverse neurological and developmental outcomes at 1 year of age was found in neonates who had evidence of seizure activity within first 48 h in the post-operative period.(Rappaport et al., 1998) Also, increased mortality was significantly associated with occurrence of post-operative seizures and delay in recovery of the aEEG background pattern beyond 48 h.(Gunn et al., 2012) The value of cEEG monitoring to assess abnormal cerebral activity in the pre and post-operative period in larger groups of neonates with CHD undergoing palliative or corrective cardiac surgery has not been previously reported. The aim of this retrospective cohort study was to assess the role of various components of cEEG in the pre-operative and post-operative period and to correlate it with abnormal neurodevelopmental outcomes at 12–24 months in neonates with congenital heart disease requiring cardiac surgery. We also investigated if there was an association between cross clamp time, arrest time and CPB time with cEEG findings after cardiac surgery and cognitive, language and motor scores on Bayley Scales of Infant and Toddler Development (BSID – III) at 12–24 months. We hypothesized that infants with CHD have abnormal pre-operative and post- operative cEEG findings and that these abnormalities were associated with poor outcomes in BSID- III scores at 12–24 months of age.

## Methods

### Study Population

This retrospective cohort study was performed at the neonatal and pediatric intensive care units (NICU and PICU) of Cleveland Clinic Children’s Hospital, Cleveland, Ohio, USA. From the medical records database, all infants that had been admitted with a diagnosis of CHD between January 2010 to December 2018 and had been monitored with cEEG were identified. In our hospital, cEEG is a routine procedure for all infants with CHD prior to and after cardiac surgery. All infants with CHD undergoing surgical repair have a pre-operative and post-operative cranial US. In the past year, our institute had 141 live births with a cardiac diagnosis (42 infants with complex cardiac anomalies, 8 infants with fetal arrythmia, 16 infants with cardiac function concerns and 75 infants with other cardiac anomalies including but not limited to VSDs, mild valvular stenosis, vascular rings, etc.). Our institute currently has three Pediatric cardiothoracic surgeons and over twenty pediatric cardiologists managing care for infants with CHD. The surgical management of CHD did not change during this period. Infants with any type of CHD who underwent cardiac surgery using CPB from birth until 44 weeks gestational age (GA) were included in the study. Neonates with gestational age less than 36 weeks, confirmed genetic disorders, multiple congenital anomalies or known underlying neurological abnormalities were excluded from the study. Infants that were transferred to another facility prior to critical care service discontinuation and infants who underwent cardiac transplantation within 30 months of cardiac surgery were also excluded. The study was approved by the Institutional Review Board (IRB) and Pediatric Institute Research Committee at Cleveland Clinic Foundation. All infants with CHD who underwent surgical correction were scheduled for outpatient follow up by pediatric neurologists to monitor neurodevelopmental outcomes in this high-risk population.

### Continuous EEG (cEEG)

A 19-channel continuous cEEG recording was performed prior to and for at least 24 h after cardiac surgery. Pre-operatively, bedside EEG monitoring (BEM) recording was obtained for 24 h typically performed 1–2 days prior to surgery. Post-operative cEEG was started after the patient was transferred to the ICU and hemodynamic stability was achieved. Only infants with pre-operative and post-operative cEEG recordings were included in this study. Twenty electrodes were placed according to the international 10–20 montage system (modified for neonates) with collodion adhesive. The cEEG service includes acquisition and review software, network infrastructure, trained and licensed EEG technologists and physicians. The Cleveland Clinic EEG laboratory is accredited by ABRET. cEEG was performed using the Nihon-Koden digital video EEG system with a portable EEG acquisition machine networked to the main server allowing EEG review at the bedside and also, remotely in the central monitoring unit.

cEEG variables such as background patterns, inter-burst interval (IBI) – amplitude and duration, symmetry, synchrony, presence of sleep wake cycles, grapho-elements and seizure activity were assessed. The cEEGs were classified as normal or abnormal based on background patterns. Normal EEG background was defined as normal continuity and discontinuity (IBI < 4 s in quiet sleep), IBI Amplitude 25–50 µV in awake or active sleep, symmetry and synchrony, spontaneous cycling among wake, active and quiet sleep and normal grapho-elements. Furthermore, severity of encephalopathy was graded as mild, mild-moderate, moderate, moderate-severe, severe encephalopathy according to classification of neonatal EEGs by Shellhaas et al., 2008, 2011 based on EEG background features including presence or lack of continuity or discontinuity, synchrony, symmetry, IBI amplitude and duration and grapho-elements seen during most part of the EEG recording. (Shellhaas et al., 2008, 2011) Sleep wake cycle was graded as normal or absent. Epileptic activity was classified as single seizure, multiple seizures or status epilepticus. Ictal discharges were characterized for timing, multifocality, lateralization and anti-seizure medication. Immediate post-operative EEG was obtained as bedside cEEG monitoring for initial 60 min post-surgery. All neonatal EEGS were interpreted by the pediatric epileptologist team for clinical purposes. For the study, cEEG recordings were assessed independently by two investigators, one a neonatologist with experience in cEEG interpretation and second, a senior EEG technician. If any discrepancies in findings were noted, final decisions were made by a pediatric epileptologist. cEEG findings were described for the entire length of available recording for each neonate.

### Bayley Scales of Infant and Toddler Development – 3rd Edition (BSID-III)

The Bayley Scales of Infant and Toddler Development, Third Edition (Bayley-III) is an individually administered instrument designed to assess the developmental functioning of infants, toddlers, and young children aged between 1 and 42 months. The Bayley-III provides coverage of the following five domains: cognitive, language, motor, adaptive, and social-emotional development. For this study, we used the three main domains – cognitive scale, language scale (includes receptive and expressive communication) and motor scale (includes gross and fine motor skills). A mean composite score of 100 was considered within normal limit, with a standard deviation of 15. A score between 70 and 85 was considered as mild delay, scores between 55 and 70 as moderate delay and scores below 55 was classified as profound delay.

### Clinical Parameters

Baseline characteristics that were recorded included demographics, birth history (gestational age, birth weight, Apgar scores, cord pH, mode of delivery), type of CHD and details pertaining to the surgery including age at surgery, pre-operative and discharge oxygen saturation, The Society of Thoracic Surgeons-European Association for Cardio-Thoracic Surgery (STAT) score, type of surgical repair, time to first chest closure, days till first extubation, extra-corporeal support days, CPB time, cross clamp time, regional cerebral perfusion time, cardiac arrest requiring cardiopulmonary resuscitation, acute kidney injury, intensive care length of stay, total length of stay, use of steroids and sedatives started in 24–48 h post operatively were also noted. Neonates were classified as Class 1 (Two ventricles with no aortic arch obstruction), Class 2 (Two ventricles with aortic arch obstruction), Class 3 (One ventricle without arch obstruction), and Class 4 (One ventricle with arch obstruction) based on the American Heart Association (AHA) anatomic classification of CHD.

### Data Collection

Data were collected and managed using Research Electronic Data Capture (REDCap), a web based electronic application through Cleveland Clinic Children’s Hospital.

### Statistical Analysis

All statistical analyses were performed using SAS 9.4 software (SAS Institute, Cary, NC). All analyses were performed on a complete-case basis and all tests were two-tailed and performed at a significance level of 0.05. Data were described using medians and ranges for continuous variables and counts and percentages for categorical variables. Associations of cEEG findings were assessed using non-parametric Kruskal-Wallis tests for categorical variables and Spearman rank correlations for continuous and ordinal findings. P- values of < 0.05 were considered to indicate statistical significance.

## Results

**Fig. 1:**
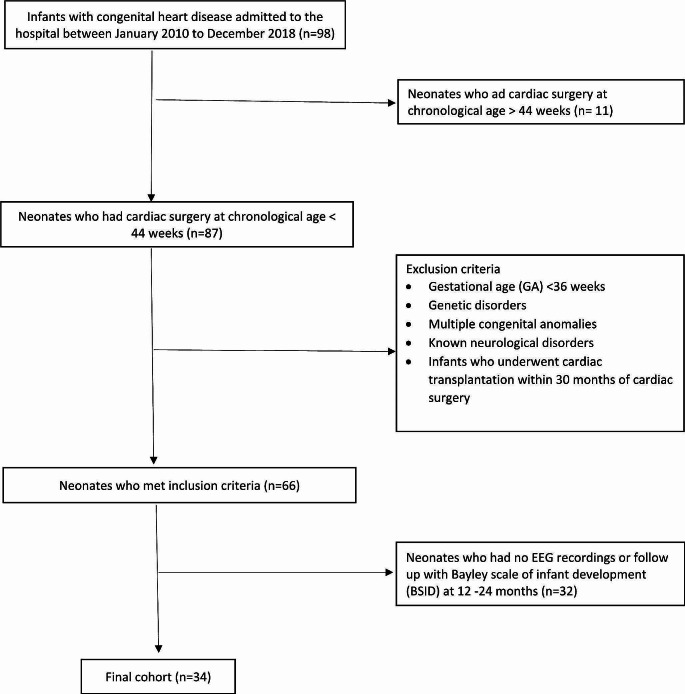
Flow diagram of the study population

A total of 98 patients with CHD who underwent surgical correction with CPB were identified. Of these 98 patients, 87 patients underwent surgery by chronological age of 44 weeks. 21 patients were excluded from the study. The reasons for not undergoing cEEG monitoring included patients transferred from outside facilities for surgical correction and critical clinical condition in the immediate post-operative period requiring repeat surgical intervention. 32 infants had no EEG recordings or BSID follow up. The final cohort consisted of 34 infants as shown in Fig. 1.

Patient demographic characteristics and neurological assessments are shown in Table 1. Of the 34 patients included in the study, 21 (62%) were males and 32 (94%) belonged to non-Hispanic ethnic group. The median gestational age was 39 weeks (IQR 36–42). Surgery in these infants took place at median age of 7 days (IQR 0–44), with median length of ICU stay of 13 days (IQR 2.0–69) and total length of stay 24 days (IQR 8.0–218). 1 out of 34 patients died in the post-operative period, with an overall mortality rate of 2.9%. All 34 neonates had neurological abnormalities during long term follow-up. Most common neurological abnormality observed was developmental delay (76%) and microcephaly (35%). Only 3 neonates had pre-operative MRI done, all of which were abnormal showing a stroke in 2 out of 3 neonates. 34 neonates had pre- and 18 neonates had post-operative head US obtained showing abnormal results in 33% of neonates after surgery (1 infant had stroke and 5 infants had other abnormalities on post-operative cranial US) (Table 1.). In this study, only 3 patients had clinical or sub clinical seizures, of which 2 patients received anti-seizure medications.

**Table 1:** Descriptive statistics, neurological status and classification of cardiac defects and surgical procedures for infants with congenital heart disease (*N* = 34)

	Total (*N* = 34)	
Factor	*N*	Statistics
Sex, n (%)	34	
Male		21 (62)
Female		13 (38)
Age at surgery (days), median (min, max)	34	7.0 (0, 44)
Ethnicity, n (%)	34	
Hispanic		2 (5.9)
Non-Hispanic		32 (94)
Gestation (Weeks), median (min, max)	34	39 (36, 42)
Head circumference percentile at birth, median (min, max)	33	36 (0.010, 100)
Weight percentile at birth, median (min, max)	34	37 (0.010, 94)
ICU length of stay, median (min, max)	34	13 (2.0, 69)
Total length of stay, median (min, max)	34	24 (8.0, 218)
Deceased, n (%)	34	1 (2.9)
Neurological status, n (%)	34	
Abnormal		34(100)
Type of neurological abnormality n (%)	34	
Developmental delay	34	26 (76)
Microcephaly	34	12 (35)
Macrocephaly	34	2 (5.9)
Stroke	34	7 (21)
Motor apraxia/Coordination	34	1 (2.9)
Behavioral/Emotional	34	2 (5.9)
Seizures	34	8 (24)
ADHD	34	2 (5.9)
Other	34	26 (76)
Pre-operative brain MRI abnormality, n (%) (MRI obtained in 3 patients)	3	
Stroke		2 (67)
Other		1 (33)
Post-operative brain MRI abnormality, n (%) (MRI obtained in 2 patients)	1	
Other		1 (100)
Pre-operative head US abnormality, n (%) (head US obtained in 34 patients)	5	
Other		5 (100)
Post-operative head US abnormality, n (%) (head US obtained in 18 patients)	6	
Stroke		1 (17)
Other		5 (83)
Class, n (%)	34	
Two ventricles with no aortic arch obstruction (Class 1)		14 (41)
Two ventricles with aortic arch obstruction (Class 2)		6 (18)
One ventricle without arch obstruction (Class 3)		6 (18)
One ventricle with arch obstruction (Class 4)		8 (24)
Type of cardiac lesion/s		
Single ventricle physiology (HLHS, DORV, DOLV)^a^, n (%)	34	14 (41)
Tetralogy of Fallot, n (%)	34	1 (2.9)
Transposition of great arteries, n (%)	34	13 (38)
AV canal, n (%)	34	1 (2.9)
Heterotaxy, n (%)	34	0 (0)
Atrial septal defect, n (%)	34	8 (24)
Ventricular septal defect, n (%)	34	6 (18)
Other, n (%)	34	26 (76)
Type of surgery, n (%)	34	
Single ventricular repair		14 (41)
Biventricular repair		20 (59)
STAT^b^ Category, n (%)	34	
1		3 (8.8)
2		2 (5.9)
3		11 (32)
4		8 (24)
5		10 (29)
Cross clamp time (minutes), median (min, max)	34	50 (0, 81)
Length of time on bypass (minutes), median (min, max)	34	117 (0, 166)
Did patient require ECMO^c^ within 7 days of surgery, n (%)	34	
No		32 (94)
Yes		2 (5.9)
Days of ECMO, n (%)	5	
2		3 (60)
3		1 (20)
4		1 (20)
Sedation, n (%)	34	
Yes		32 (100)
Paralysis, n (%)	34	
No		30 (88)
Yes		4 (12)

Statistics presented as Median (min, max), N (column %).

^a^ HLHS- Hypoplastic left heart syndrome, DORV- Double outlet right ventricle, DOLV- Double outlet left ventricle

^b^ The Society of Thoracic Surgeons-European Association for Cardio-Thoracic Surgery

^c^ ECMO- Extracorporeal membrane oxygenation

The cardiac defects were Class 1 in 14 neonates (41%), Class 2 in 6 neonates (18%), Class 3 in 6 neonates (18%) and Class 4 in 8 neonates (24%) as shown in Table 1. The median duration of CPB was 117 min (IQR 0-166) and cross clamp time was 50 min (IQR 0–81). Two neonates (5.9%) were placed on ECMO with 20% requiring ECMO for 2 days and 20% neonates for 3 days. Both the neonates requiring ECMO had normal pre and post-operative cranial US findings, abnormal pre-operative EEG findings (mild encephalopathy) and post-operative EEG findings (moderate-severe encephalopathy). One of the infants who required ECMO with single ventricular physiology had seizures and developmental delay noted during BSID-III assessment (low cognitive, language and motor composite scores).

### Neurodevelopmental Assessment Scores

Overall neurocognitive outcomes for the cohort assessed by BSID -III are described in Table 2. Bayley Scales of Infant and Toddler Development, Version 3 was administered at a median age of 17 months (IQR 12–24). For the cognitive subtest, median scaled score was 8 (IQR 1.0–15). Median cognitive composite score was 90 (IQR 55–125). 13 neonates (38%) had an average cognitive score, where as 21% had a low average score and 12% had extremely low composite score. Median language composite score was 83 (IQR 47–135) with 9 neonates (27%) scoring an average score and 24% with an extremely low score. On the motor subtest, median composite score was 78 (IQR 46–124), 26% of these neonates had an average, borderline and extremely low scores respectively.

**Table 2:** Descriptive statistics for neurodevelopmental outcomes at 12–24 months

	Total (*N* = 34)	
Factor	*N*	Statistics
Age at BSID^a^ (months), median (min, max)	34	17 (12, 24)
BSID Cognitive Scaled, median (min, max)	34	8.0 (1.0, 15)
BSID Cognitive Composite, median (min, max)	34	90 (55, 125)
BSID Cognitive Percentile, median (min, max)	34	25 (0.10, 95)
BSID Cognitive Category, n (%)	34	
Extremely Low		4 (12)
Borderline		3 (8.8)
Low Average		7 (21)
Average		13 (38)
High Average		4 (12)
Superior		3 (8.8)
BSID Language Scaled, median (min, max)	33	14 (2.0, 32)
BSID Language Composite, median (min, max)	33	83 (47, 135)
BSID Language Percentile, median (min, max)	33	13 (0.10, 99)
BSID Language Category, n (%)	33	
Extremely Low		8 (24)
Borderline		7 (21)
Low Average		7 (21)
Average		9 (27)
High Average		1 (3.0)
Very Superior		1 (3.0)
BSID Motor Scaled, median (min, max)	34	13 (2.0, 28)
BSID Motor Composite, median (min, max)	34	78 (46, 124)
BSID Motor Percentile, median (min, max)	34	6.5 (0.10, 95)
BSID Motor Category, n (%)	34	
Extremely Low		9 (26)
Borderline		9 (26)
Low Average		5 (15)
Average		9 (26)
High Average		1 (2.9)
Superior		1 (2.9)

Statistics presented as Median (min, max), N (column %).

^a^ BSID Bayley Scale of Infant Development

### Correlation of cEEG Components with BSID-III Subtests

We examined the association of various components of EEG background activity with subtests of BSID-III – cognitive, language and motor (Table 3.). Pre-operative inter-burst interval amplitude and duration, immediate and 24-hour post-operative cEEG background activity including continuity, excessive discontinuity, synchrony, inter-burst interval amplitude and duration and presence or absence of sleep wake cycles were not associated with poor outcomes in the cognitive and motor subtests of BSID- III (Table 3.).

**Table 3:** Correlation of cEEG with cognitive, language and motor composite score (CS) at 12–24 months

	Cognitive composite score				Language composite score			Motor composite score		
Factor	*N*	Statistics		*P* value	*N*	Statistics	*P* value	*N*	Statistics	*P* value
Pre-op EEG Interburst Interval - Amplitude	32	0.09 (-0.27, 0.43)		0.62	32	-0.06 (-0.40, 0.30)	0.76	32	0.04 (-0.32, 0.38)	0.84
Pre-op EEG Interburst Interval – Duration	32	-0.08 (-0.42, 0.28)		0.67	32	-0.04 (-0.39, 0.32)	0.85	32	-0.13 (-0.46, 0.23)	0.48
Post-op EEG Continuous	32			0.26	32		0.066			0.45
No	17	95 [80, 110]			17	89 [77, 103]		17	82 [67, 97]	
Yes	15	90 [75, 95]			15	77 [62, 94]		15	73 [70, 91]	
Post-op EEG Discontinuous	32			0.21	32		0.065			0.54
No	14	90 [75, 95]			14	76 [62, 94]		14	76 [70, 91]	
Yes	18	95 [80, 110]			18	88 [77, 103]		18	82 [67, 97]	
Post-op EEG Synchronous	32			0.37			0.27			0.60
No	11	90 [80, 95]			11	83 [59, 94]		11	73 [64, 97]	
Yes	21	95 [80, 110]			21	83 [74, 103]		21	82 [73, 97]	
Post-op EEG Interburst Interval - Amplitude	32	0.11 (-0.25, 0.44)		0.56	32	0.01 (-0.34, 0.36)	0.95	32	0.01 (-0.34, 0.36)	0.96
Post-op EEG Interburst Interval - Duration	32	0.28 (-0.07, 0.58)		0.12	32	0.34 (-0.00, 0.62)	0.053	32	0.22 (-0.14, 0.53)	0.22
Post-op 24 H EEG Continuous	29			0.45	29		0.096			0.59
No	17	95 [80, 110]			17	97 [65, 103]		17	85 [64, 97]	
Yes	12	90 [83, 95]			12	78 [74, 83]		12	75 [70, 85]	
Post-op 24 H EEG Discontinuous	29			0.45	29		0.096			0.59
No	12	90 [83, 95]			12	78 [74, 83]		12	75 [70, 85]	
Yes	17	95 [80, 110]			17	97 [65, 103]		17	85 [64, 97]	
Post-op 24 H EEG Sleep-wake cycle	29			0.079	29		0.11			0.36
No	19	95 [80, 110]			19	89 [74, 103]		19	82 [67, 100]	
Yes	10	90 [65, 95]			10	76 [65, 83]		10	76 [64, 88]	
Post-op 24 H EEG Interburst Interval - Amplitude	29	-0.14 (-0.48, 0.24)		0.48	29	-0.34 (-0.63, 0.03)	0.070	29	-0.17 (-0.51, 0.21)	0.37
Post-op 24 H EEG Interburst Interval - Duration	29	0.20 (-0.18, 0.53)		0.31	29	0.33 (-0.04, 0.62)	0.082	29	0.10 (-0.27, 0.45)	0.60
Severity of pre-op EEG	32	0.01 (-0.34, 0.36)		0.95	32	0.12 (-0.24, 0.46)	0.51	32	0.00 (-0.35, 0.35)	0.99
Severity of post-op EEG	32	0.09 (-0.27, 0.42)		0.64	32	0.12 (-0.24, 0.45)	0.51	32	0.02 (-0.33, 0.37)	0.90
Severity of post-op 24 H EEG	29	0.19 (-0.19, 0.52)	0.32		29	0.41 (0.05, 0.68)	***0.026***	29	0.16 (-0.22, 0.50)	0.41
Cross- clamp time	32	0.04 (-0.25, 0.33)	0.78		32	0.32 (0.02, 0.56)	***0.036***	32	0.08 (-0.22, 0.36)	0.62
Arrest time	32	-0.00 (-0.30, 0.30)	0.99		32	0.13 (-0.19, 0.42)	0.43	32	-0.21 (-0.48, 0.10)	0.18
Bypass time	32	-0.05 (-0.33, 0.24)	0.75		32	0.24 (-0.06, 0.50)	0.12	32	-0.05 (-0.33, 0.24)	0.75

Statistics presented as Median [25th, 75th percentiles] with Kruskal-Wallis test or Spearman’s correlation (95% CI).

### Correlation of cEEG and Cognitive and Motor Subtests of BSID-III

Pre-operative and immediate and 24 h post-operative severity of EEG findings and encephalopathy as well as duration of cross- clamp time, bypass time and arrest time were not associated poor cognitive or motor BSID-III scores at 12–24 months of age.

### Correlation of cEEG and Language Subtest of BSID-III

As shown in Table 3. Pre-operative inter-burst interval amplitude and background, immediate and 24 h post-operative background activity components on cEEG did not correlate with poor outcomes in the language subtest. Severity of EEG classification and encephalopathy for the 24 h post-operative EEG was associated with a lower language composite score and correlated with poor performance in the language subtest of BSID-III (*p* = 0.026). Also, longer duration of cross clamp time was associated with below average language composite scores during BSID-III testing at 12–24 months (*p* = 0.036).

Degree of functional myocardial dysfunction in this cohort was assessed using clinical evaluation tool – Vasoactive -inotropic score (VIS). Max VIS (median) at 24 h and 48 h post-operatively was 8.5 and 6.0 respectively. Higher VIS was associated with lack of sleep wake cycling in the post-operative period (p 0.024), longer duration of CPB (p 0.013), longer duration on IBI amplitude (p 0.017) and severity of encephalopathy (*p* < 0.001) during pre-operative EEG recording (Table 4.). There was so significant association of VIS post-operatively with neurodevelopmental outcomes assessed by BSID-III in this cohort.

**Table 4:** Vasoactive inotropic score (VIS) at 24 h post op vs. EEG

Factor	*N*	Statistics	*p*-value
Post-op 24 H EEG Continuous			0.30
No	17	9.0 [5.0, 15]	
Yes	12	15 [6.0, 20]	
Post-op 24 H EEG Discontinuous			0.30
No	12	15 [6.0, 20]	
Yes	17	9.0 [5.0, 15]	
Post-op 24 H EEG Sleep-wake cycle			***0.024***
No	19	14 [7.0, 22]	
Yes	10	5.0 [2.5, 13]	
Length of Time on Bypass (Minutes)	34	0.42 (0.09, 0.66)	***0.013***
Circulatory Arrest Time (Min)	34	0.28 (-0.07, 0.57)	0.12
Cross Clamp Time (Mins)	34	-0.02 (-0.35, 0.32)	0.92
Post-op 24 H EEG Interburst Interval - Amplitude	29	-0.28 (-0.59, 0.09)	0.14
Post-op 24 H EEG Interburst Interval - Duration	29	0.32 (-0.05, 0.62)	0.087
Pre-op EEG Interburst Interval - Amplitude	32	-0.42 (-0.67, -0.08)	***0.017***
Pre-op EEG Interburst Interval - Duration	32	0.25 (-0.11, 0.55)	0.17
Severity of post-op EEG	32	-0.18 (-0.49, 0.18)	0.34
Severity of post-op 24 H EEG	29	0.06 (-0.31, 0.42)	0.75
Severity of pre-op EEG	32	0.68 (0.44, 0.83)	***< 0.001***

Statistics presented as Median [25th, 75th percentiles] with Kruskal-Wallis test or Spearman’s correlation (95% CI).

## Discussion

This study evaluated the role of cEEG background patterns, presence of sleep wake cycles and seizure activity in the pre-operative and post-operative period and its correlation to abnormal neurodevelopmental outcomes at 12–24 months in neonates with CHD requiring cardiac surgery. In this retrospective study, we report that (1) majority of infants with CHD that underwent surgical repair by 44 weeks GA had below average scores in all three subtests (cognitive, language and motor) of BSID-III at 12–24 months of age (2) Severity of encephalopathy classification for 24-hour postoperative EEG was correlating with poor language composite scores during BSID-III) Longer duration of cross-clamp time was associated with below average language composite score.

Our results demonstrated a higher rate (100%) of neurological abnormalities observed among the thirty- four neonates that were included in the study and subsequently followed-up in the outpatient clinic. Most common neurological abnormality in this study population was abnormal tone and developmental delay (76%) followed by microcephaly (35%). Previous studies have demonstrated that more than 50% of newborns with complex heart defects have clinical evidence of neurological impairments on exam before surgery and these are subsequently risk factors for long term neurodevelopmental abnormalities. (Gaynor et al., 2006; Hövels-Gürich et al., 2002; Limperopoulos et al., 1999) Another study looking at 2 different forms of CPB for correction of TGA showed neurological impairment in up to 37% of enrolled patients. (Bellinger et al., 1995) A higher rate of neurological abnormalities observed among neonates compared to previous studies could be due to the smaller sample size and nature of cardiac defects of neonates included in this study. In our study, 67% of neonates with abnormal pre-operative MRI demonstrated a finding of stroke. These findings are similar to results demonstrated in previous studies. (Khalil et al., 2014; Latal et al., 2015; Licht et al., 2004; Mahle et al., 2002; McQuillen et al., 2007)

In this study population, median cross clamp time and CPB time was 50 min and 118 min respectively. Prolonged circulatory arrest time is a major risk factor for neurodevelopmental impairments. (Bellinger et al., 1995; Limperopoulos et al., 2002) Follow-up studies in children who had undergone open heart surgery with periods of arrest longer than 60 min and as short as 45 min showed associated neurodevelopmental impairments. ^50,51^ In our cohort, longer duration cross-clamp time was associated with poor language composite score on BSID-III assessment at 12–24 months of age. Some previous studies including the Boston circulatory Arrest Trial have provided evidence for the deleterious effects of deep hypothermic circulatory arrest vs. low flow CPB (Bellinger et al., 2003; H.H. et al., 2002; Hovels-Gurich et al., 1997).

Factors contributing to neurologic and developmental impairment in infants with CHD are multifactorial and comprises a complex interaction between preoperative, intraoperative and postoperative factors. (Majnemer & Limperopoulos, 1999) Also, neurodevelopmental dysfunction rates vary by disease complexity. (Martinez-Biarge et al., 2013) In our cohort, BSID-III was administered at a median age of 17 months. The median cognitive composite score was 90 and 21% neonates had below average cognitive scores. The median motor composite score was 78 and 67% of these neonates had below average scores. Median language composite score was 83 with 66% of neonates demonstrating below average scores. Previous studies have demonstrated that mean composite scores for Bayley III were slightly higher compared with composite scores for Bayley II. (Mebius et al., 2017) Mean cognitive scores ranged from 91.0 to 104.8, mean language scores ranged from 87.8 to 97.0 and mean motor scores ranged from 86.0 to 97.0. (Gunn et al., 2012a, b; Andropoulos et al., 2012) Masoller et al. in a case-control stud demonstrated lower cognitive, language and motor. (Masoller et al., 2016) Andropoulos et al. demonstrated composite scores for cognition (102 $\pm$ 13.3), motor (89.6 $\pm$ 14.1) and language (87.8$\pm$ 12.5) and no association between preoperative brain injury and neurodevelopmental outcomes. (Andropoulos et al., 2014)

EEG background activity has been shown to be an important measure of functional brain maturation and impairment. (Lamblin et al., 1999) This study demonstrated that various components of cEEG background activity during pre, immediate and 24-hour postoperative period was not associated with poor median cognitive composite, language and motor composite scores. Severity of encephalopathy during pre, immediate and 24-hour postoperative EEG tracing was not correlating with lower cognitive and motor composite scores. This is consistent with findings of Gunn et al. demonstrating no association between preoperative aEEG background pattern and neurodevelopmental outcomes. (Gunn et al., 2012) Similarly, Robertson et al. showed no association between EEG abnormalities, reduced cerebral blood flow and neurodevelopmental outcomes. (Robertson et al., 2004) Severity of encephalopathy during the 24 h postoperative period was associated with poor language composite score on BSID-III (*p* = 0.026). Andropoulos et al. reported lower language scores associated with preoperative brain injury on MRI in infants with TGA who underwent cardiac surgery. (Andropoulos et al., 2012) Another study by William et al. demonstrated a positive correlation between preoperative left frontal polar and left frontal ß power and cognitive scores. (Williams et al., 2012) Hence, neonates with higher grades of encephalopathy and severity on EEG in the immediate 24 h postoperative period are at a higher risk of poor language scores on BSID-III.

This study has strengths and limitations. This study used continuous conventional 10-20system EEG for neuromonitoring of neonates with CHD. Secondly, the study assessed various components of EEG findings and used this data to classify EEG background based on severity of encephalopathy. However, this study has limitations too. This is a retrospective cohort study and included a small number of patients. The small number of participants did not allow for control of the severity and etiology of encephalopathy and future research is required to investigate this further. Only few neonates included in the study had brain MRI obtained in pre and post-operative period. Approximately 50% of neonates with CHD who underwent cardiac surgery were lost to follow up and did not have Bayley assessment at 12–24 months of age. Although Bayley Scales of Infant and Toddler Development-III provides an accurate assessment of a child’s developmental status at 12–24 months, it has limited predictive value for later outcomes like intelligence quotient (IQ) or behavioral issues in adolescence. This cohort study included a wide spectrum of CHD types having different cEEG abnormalities further preventing subgroup analysis for associations between class of cardiac defects and cEEG findings and neurodevelopmental outcomes.

## Conclusion

In this heterogeneous group of neonates with CHD who underwent repair by 44 weeks GA, majority of neonates had below average cognitive, language and motor composite scores on BSID-III at 12–24 months of age. Longer duration of cross-clamp time was specifically associated with poor language composite scores. It is interesting to report a significant association between severity of encephalopathy during 24 h postoperative EEG monitoring with poorer language composite scores on BSID-III assessment. Early identification of high- risk infants is essential so that these infants would benefit from targeted early interventions to minimize neuromotor and behavioral deficits in the future. Further studies are needed to assess relationship between EEG background findings and neurodevelopmental outcomes in pre-school and school aged children with CHD.
